# Social and cultural aspects of 'malaria' and its control in central Côte d'Ivoire

**DOI:** 10.1186/1475-2875-7-224

**Published:** 2008-10-30

**Authors:** Clémence Essé, Jürg Utzinger, Andres B Tschannen, Giovanna Raso, Constanze Pfeiffer, Stefanie Granado, Benjamin G Koudou, Eliézer K N'Goran, Guéladio Cissé, Olivier Girardin, Marcel Tanner, Brigit Obrist

**Affiliations:** 1 Institut d'Ethno Sociologie, Université de Cocody-Abidjan, 01 BP V34, Abidjan 01, Côte d'Ivoire; 2 Centre Suisse de Recherches Scientifiques, 01 BP 1303, Abidjan 01, Côte d'Ivoire; 3 Department of Public Health and Epidemiology, Swiss Tropical Institute, P.O. Box, CH-4002, Basel, Switzerland; 4 Division of Epidemiology and Social Medicine, School of Population Health, University of Queensland, Herston Road, Brisbane, QLD 4006, Australia; 5 Molecular Parasitology Laboratory, Queensland Institute of Medical Research, 300 Herston Road, Brisbane, QLD 4006, Australia; 6 Unité de Formation et de Recherche des Biosciences, Université de Cocody-Abidjan, 22 BP 770, Abidjan 22, Côte d'Ivoire; 7 Fondation Rurale Interjurassienne, Courtemelon, P.O. Box 65, 2852 Courtételle, Switzerland

## Abstract

**Background:**

A sound local understanding of preventive measures and health-seeking behaviour is important for the effective control of malaria. The purpose of this study was to assess the knowledge, attitudes, practices and beliefs of 'malaria' and its control in two rural communities of central Côte d'Ivoire, and to examine associations between 'malaria' and the households' socioeconomic status.

**Methods:**

A cross-sectional household survey was carried out, using a combination of qualitative and quantitative methods. People's socioeconomic status was estimated, employing a household asset-based approach.

**Results:**

Malaria was identified as *djèkouadjo*, the local folk name of the disease. Although people were aware of malaria-related symptoms and their association with mosquitoes, folk perceptions were common. In terms of treatment, a wide array of modern and traditional remedies was employed, often in combination. Individuals with a sound knowledge of the causes and symptoms of malaria continued to use traditional treatments and only a few people sleep under bed nets, whereas folk beliefs did not necessarily translate into refusal of modern treatments. Perceived causes of malaria were linked to the household's socioeconomic status with wealthier individuals reporting mosquitoes more frequently than poorer households. Bed nets were more frequently used in wealthier social strata, whereas other protective measures – perceived to be cheaper – were more prominent among the poorest.

**Conclusion:**

Equitable access to resources at household, community and health system levels are essential in order to enable community members to prevent and treat malaria. There is a need for community-based approaches that match health care services with poor people's needs and resources.

## Background

Malaria is a devastating vector-borne disease that primarily occurs in the developing world. Over two billion people are at risk of malaria and, in 2001, an estimated 1.2 million people died from the disease [1]. There were an estimated 515 million clinical episodes due to an infection with *Plasmodium falciparum *in 2002 [2]. African governments spend more than 1% of their gross domestic product (GDP) to combat malaria and the estimated annual direct and indirect costs attributable to malaria in sub-Saharan Africa are in excess of US$12 billion [3].

In Côte d'Ivoire, malaria remains of the most pressing public health issues. Due to the climatic and geographic conditions, the disease is endemic in all parts of the country and transmission occurs all year round. Malaria accounts for more than 40% of in-patient and out-patient attendances in health care delivery structures and an estimated 20% of in-patient mortality [4]. Recent studies carried out in central Côte d'Ivoire showed that high malaria transmission rates in the face of limited personal protection result in a significant number of work days lost among rural farmers and have a negative impact on livelihood and economic revenues [5,6]. Studies also identified a need for improving malaria control measures by taking into account local beliefs and practices. This issue underscores the growing body of literature on social and cultural aspects of malaria [7-10]. Indeed, previous work has shown the relevance of people's illness classification, the recognition of symptoms, as well as explanation of causes and help-seeking behaviour for improvements in malaria control. For example, a deeper understanding of illness experiences at a household level can enhance appropriate home management of malaria and more effective use of insecticide-treated nets (ITNs) [11].

It is also important to note that people's experience and management of malaria, for example the use of ITNs, is influenced by socioeconomic factors [12]. Malaria is both a main cause and consequence of poverty [13-17]. People suffering from frequent episodes of clinical malaria are economically less productive, and poor people are more likely to fall ill from malaria. Moreover a study in western Côte d'Ivoire has shown that the poorest people are least likely to benefit from health interventions and adequate service delivery [18].

Here, results from a household-based survey, carried out in two rural communities living in central Côte d'Ivoire, are reported. The study complements recent investigations in the same region, focusing on agricultural, biomedical and entomological issues of malaria [5,6,19]. The following four questions governed the research. First, does the disease 'malaria' correspond to an illness recognized by the villagers? Second, are villagers aware of a link between malaria-related illness and mosquitoes? Third, what are the main determinants of villagers' help-seeking? Fourth and finally, do villagers know and use methods to prevent malaria?

## Methods

### Framework of the study

**Figure 1:**
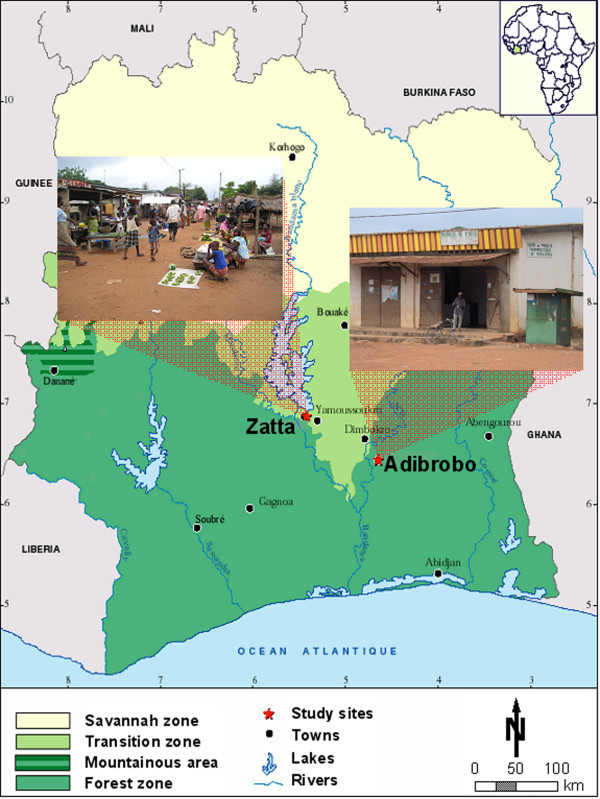
**Map of Côte d'Ivoire with the two study sites in the central part of the country**. Superimposed are two photographs, one depicting a typical market scene in Zatta, and the other showing a public pharmacy in Adibrobo.

Between 1999 and 2003, a rural development project operating at the interface of agriculture, health and wealth was implemented in Adibrobo (a small settlement located near Tiémélékro, south-east of Yamoussoukro), and Zatta (a village situated north-west of Yamoussoukro) [5,6]. Figure 1 shows the location of the two study villages in central Côte d'Ivoire, including snapshots of typical environments and activities of local residents. The project's aim was to increase agriculture-based income through the implementation of small-scale intensive vegetable production. An initial exploratory survey in the villages revealed that malaria, diarrhoeal diseases (epidemics in 1997) and respiratory infections were the most pressing public health problems. Subsequent studies included a cross-sectional survey of malaria parasite rates among school-aged children (prevalence rates of 87% and 72% were found in Zatta and Adibrobo, respectively), entomological surveys [6,19] and community awareness campaigns promoting the use of ITNs. The findings called for locally adapted measures to prevent and control malaria with the aim to reduce farmers' vulnerability, which in turn might result in increased agricultural productivity and economic revenues.

According to the 1998 census, Adibrobo has a population of 700 inhabitants. The nearest health centre is in Tiémélékro, located 7 km west, which is run by a medical doctor, two assistant nurses and a midwife. There is one primary school, and villagers have access to running water and are connected to the power grid. The population size of Zatta in 1998 was 3,315 inhabitants. There is a dispensary staffed with one nurse, three assistant nurses and one midwife. There are three primary schools. Villagers have running water at home, and they are connected to the power grid. Small multi-purpose dams were constructed in both villages in the mid-1970s. In addition, since the late 1990s, there is irrigated rice farming in Zatta, but not in Adibrobo [6,19].

### Cross-sectional household-based survey

Enumeration of households in May 2002 revealed a total of 476 households in Zatta and 110 in Adibrobo. In Zatta, approximately a third of the households were randomly selected (n = 176). Due to the small overall number of households in Adibrobo a larger proportion was sampled than in Zatta, i.e. almost 60% (n = 64). A cross-sectional epidemiological design was adopted and a team, comprising of a supervisor, two field assistants (sociology students speaking the local languages), three local enumerators and two local guides, carried out the household-based surveys.

Three different data collection methods were employed, using quantitative methods that were cross-checked and complemented by qualitative methods [20]. To cover different perceptions in the community, various groups were interviewed. First, a pre-tested questionnaire was administered to male or female household heads or, in case of their absence, to respective adults in the selected households. Household heads were selected based on the assumption that they influence health-related perceptions and behaviour in their families. Second, four focus group discussions (FGDs) in two out of the three primary schools in Zatta, and two FGDs in the primary school in Adibrobo were carried out. For each FGD, eight schoolchildren (four girls and four boys, aged 10 to 14 years), were selected either from grade 5 (three FGDs) or grade 6 (three FGDs). The aim of the FDGs was to learn about children's knowledge and attitudes of malaria as well as about the influence of health education. Third, key informant interviews were carried out with representatives practicing traditional or modern medicine, health facility staff (Zatta: n = 4, Adibrobo: n = 3) and local healers (Zatta: n = 2, Adibrobo: n = 2). Local healers were identified during the cross-sectional survey, by asking the heads of household about the name of healers and address details. In Zatta, additionally, four local community leaders, mainly religious leaders, were interviewed.

During FGDs and key informant interviews, two different types of interview guides were used that were developed by the research team for community members as well as schoolchildren. The cross-sectional survey, the semi-structured guides for the FGDs and interviews were pre-tested in a neighbouring village and a primary school in Adiopodoumé, located 17 km west of Abidjan, the economic capital of Côte d'Ivoire. Besides the FGDs with schoolchildren that were conducted in French, all other qualitative interviews were done in the local languages (i.e. Agni and Baoulé). Subsequently, interviews were transcribed and translated into French.

### Data management and statistical analysis

FGDs and key informant interviews were recorded and transcribed by the field assistants into a text programme (Microsoft Word) and then converted into a software for qualitative data analysis (Maxqda version 1, VERBI Software Consult; Berlin, Germany). Text segments were coded and later compared within and across sub-samples to identify similarities and differences in answers to the four research questions.

Questionnaire data were double entered and checked for consistency with EpiInfo version 6.04 (Centers for Disease Control and Prevention; Atlanta, USA). Statistical analyses were performed with STATA software version 8.0 (STATA Corporation; College Station, USA).

The socioeconomic status of a household was determined by an asset-based approach initially developed in India [21] and successfully used in different African settings, including rural Côte d'Ivoire [15,18,22,23]. Through principal component analysis (PCA), the weights for each household asset were determined, and calculated as compound asset index following instructions from the World Bank's HNP/Poverty Thematic Group [12,24]. The first principal component (PC) explained 20.1% of the variability in the 16 variables. The greatest weights were for access to the power grid at home (eigenvector 0.44), living in a house with cement brick walls and a corrugated iron-sheet roof (0.35) and ownership of a television (0.32). The lowest scores were for livestock keeping (0.04).

Equity was assessed by dividing the resulting compound asset score into quintiles, so that each household was categorized as poorest, very poor, poor, less poor and least poor. Variables from the household study were then associated with the resulting socioeconomic strata. Concentration indices (CIs) with respective standard errors (SEs) were calculated for people's knowledge of, and practice for, prevention of malaria in relation to their socioeconomic status. Details about how to calculate the CI have been presented elsewhere [18,25]. An important feature of the CI is that it allows examining the direction of an association.

### Ethical considerations

During data collection and subsequent analyses, all relevant ethical issues that are required in social science researches were respected. In brief, all individuals who were involved in this project were informed about the purpose and procedures of the study, and about their rights as informants before they were asked for informed oral consent. Participation was entirely voluntary and each individual was free to leave the study at any time.

## Results

### Characteristics of the household sample

With the exception of ethnic origin, the demographic profile of the household heads interviewed in Zatta and Adibrobo is similar (Table 1). Baoulé is the predominant ethnic group in Zatta (79%), and Agni in Adibrobo (78%). Both groups are part of the Akan speaking ethnic supragroup of Côte d'Ivoire and Ghana. Whilst the same number of males and females were interviewed in Adibrobo (n = 32 each), slightly more females responded in Zatta (90 *vs*. 78). The majority of the respondents were Christians (Zatta: 60%, Adibrobro: 84%) and either illiterate or having completed primary education only. Household heads with secondary education included teachers (five in Zatta and six in Adibrobo). Four-fifth of the interviewees were farmers, whereas the remaining household heads earned a living as craftsman (Zatta: 11%, Adibrobo: 2%), cotton factory workers (Zatta: 5%, Adibrobo: 9%) or retailers (Zatta: 6%, Adibrobo: 5%).

**Table 1:** Characteristics of the study populations in Zatta and Adibrobo, central Côte d'Ivoire

Variables	Zatta (n = 168)	Adibrobo (n = 64)	Total (n = 232)
Ethnic group			
Baoulé	133 (79%)	7 (11%)	140 (60%)
Agni	3 (2%)	50 (78%)	53 (23%)
Dioula	17 (10%)	0	17 (7%)
Others	15 (9%)	7 (11%)	22 (10%)
Gender			
Male	78 (46%)	32 (50%)	110 (47%)
Female	90 (54%)	32 (50%)	122 (53%)
Age (years)			
15–29	49 (29%)	14 (22%)	63 (27%)
30–40	69 (41%)	19 (30%)	88 (38%)
>40	50 (30%)	31 (48%)	81 (35%)
Religion			
Christian	101 (60%)	54 (84%)	155 (67%)
Muslim	25 (15%)	1 (2%)	26 (11%)
Local religions	37 (22%)	9 (14%)	46 (20%)
Others	4 (3%)	0	4 (2%)
Education			
Illiterate	81 (49%)	34 (53%)	115 (50%)
Primary school	52 (32%)	13 (20%)	65 (28%)
Secondary school	32 (20%)	17 (27%)	49 (22%)
Profession			
Farmers	132 (78%)	54 (84%)	186 (80%)
Craftsman	19 (11%)	1 (2%)	12 (5%)
Cotton factory worker	8 (5%)	6 (9%)	14 (6%)
Retailer	9 (6%)	3 (5%)	20 (9%)

### Malaria-related illness concepts

The main ethnic groups in Zatta and Adibrobo used similar terms to refer to mild forms of malaria: "*djèkouadjo*" (Baoulé) and "*djékadjo*" (Agni). In the remainder of this paper the Baoulé term is used as an umbrella for both. Interviewees used a closely related concept for more severe manifestations of the disease "*ewueŋgo*", which mainly describes a deep yellow colouring of the palm, eyes and urine. Still another term, i.e. "*anumą*", is used for convulsion, which is considered by the villagers as a childhood health problem, and is not attributed to malaria. Interviewees feel that only traditional healers can successfully treat convulsions, a belief that is even confirmed by a nurse assistant working at the dispensary of Zatta: "*My point of view is that modern medicine can do nothing to treat convulsions. It must be treated by traditional healers*".

### Symptoms of *djèkouadjo*

As shown in Table 2, household heads use *djèkouadjo *for a cluster of symptoms representing observed physical or behavioural changes. The level of knowledge regarding malaria symptoms was high, and differences according to ethnic groups, religions and educational levels were minimal. There were only few differences between Zatta and Adibrobo. Fever was clearly the most important symptom (Zatta: 71%, Adibrobo: 69%). Another common symptom was loss of appetite. Approximately a third of the respondents in both villages mentioned the categories "yellow eyes" and "yellow urine" as signs of malaria. Jaundice, which is manifested by yellow eyes and yellow urine, is common in cases of severe *P. falciparum *malaria [26]. Additionally, most of the key informants mentioned abdominal discomfort and diarrhoea, fever, loss of appetite, weight loss, white palm and tiredness. Finally all modern health care providers also indicated constipation, diarrhoea, nausea, body aches and pains.

**Table 2:** Villagers' knowledge and practice related to *djèkouadjo *in Zatta and Adibrobo, central Côte d'Ivoire

Variable	Total	Village			
		
		Zatta	Adibrobo	χ^2^	*P*-value
Symptoms					
Fever	163 (70%)	119 (71%)	44 (69%)	0.1	0.75
Loss of appetite	112 (48%)	90 (54%)	22 (34%)	6.8	0.009
Headache	79 (34%)	63 (36%)	16 (25%)	3.2	0.073
Yellow eyes	75 (32%)	54 (32%)	21 (33%)	0.0	0.922
Yellow urine	66 (28%)	48 (29%)	18 (28%)	0.0	0.946
Vomiting	29 (13%)	22 (13%)	7 (11%)	0.2	0.657
Perceived causes					
Sun	171 (74%)	126 (75%)	45 (70%)	0.5	0.469
Mosquitoes	150 (65%)	118 (70%)	32 (50%)	8.3	0.004
God	16 (7%)	12 (7%)	4 (6%)	0.0	0.960
Sorcerer	13 (6%)	13 (8%)	0	3.9	0.048
Ancestor	6 (3%)	6 (4%)	0	1.1	0.285
Actors approached for problem solving					
Family	187 (80%)	149 (89%)	38 (59%)	25.5	<0.001
Individual	42 (18%)	25 (15%)	17 (27%)	4.3	0.039
Village	25 (11%)	10 (6%)	15 (23%)	14.7	<0.001
Traditional treatment prescribed by					
Relatives	100 (46%)	82 (52%)	18 (30%)	8.6	0.003
Traditional healer	59 (27%)	47 (30%)	12 (20%)	2.2	0.141
Self-medication	56 (30%)	29 (19%)	27 (45%)	15.7	<0.001
Friend	21 (10%)	15 (10%)	6 (10%)	0.0	0.921
Preferred treatment of the sick person					
Traditional	136 (59%)	107 (64%)	29 (45%)	6.5	0.011
Modern	76 (33%)	46 (27%)	30 (47%)	8,0	0.005
Don't know	16 (7%)	11 (7%)	5 (8%)	0.1	0.734
Diseases caused by mosquitoes					
Malaria	206 (89%)	155 (93%)	51 (80%)	8.3	0.004
Pimples (wheals)	130 (56%)	103 (62%)	27 (42%)	6.9	0.009
Fever	91 (39%)	67 (40%)	24 (38%)	0.1	0.740
Itching	83 (36%)	66 (39%)	17 (25%)	3.3	0.071
Does not know	8 (3%)	6 (4%)	2 (3%)	0.1	0.813
Measures against mosquitoes					
Removal of vegetation	137 (60%)	108 (65%)	29 (46%)	6.6	0.010
Removal of stagnant water	106 (46%)	87 (52%)	19 (30%)	8.9	0.003
Use of insecticides	55 (24%)	52 (31%)	3 (5%)	17.5	<0.001
Bush fire	18 (8%)	18 (11%)	0	5.9	0.014

### Aetiology of *djèkouadjo*

The main reported causes of *djèkouadjo *are summarised in Table 2. According to the household heads interviewed these causes were the sun (Zatta: 75%, Adibrobo: 70%) and mosquitoes (Zatta: 70%, Adibrobo: 50%), regardless of ethnic groups and religions. With regard to the sun or mosquitoes as causes of *djèkouadjo*, an interesting trend could be observed: illiterate people mentioned the sun more frequently (Zatta: 79%, Adibrobo: 82%) than those who attended primary school (Zatta: 74%, Adibrobo: 77%) and had a secondary school education (Zatta: 67%, Adibrobo: 38%). Conversely, more educated people referred to mosquitoes as a cause of malaria more often (Zatta: 88%, Adibrobo: 81%) than illiterate individuals (Zatta: 58%, Adibrobo: 29%). Among the Baoulé, some respondents also mentioned beings with special powers as causes, namely God, sorcerers or ancestors.

The following quotes illustrate the aetiology of *djèkouadjo *as described by a traditional healer in Adibrobro: "*Working for a long time under the sun leads to djékouadjo, because the blood becomes warm. God also sends djèkouadjo*", and in Zatta: "*Malaria is caused by mosquitoes and unhygienic surroundings in the household. Therefore, we need modern medicine and better hygiene for its treatment*".

All key informants put forward additional causes related to the environment, such as depositing household litter, waste water, uncleared vegetation, living in close proximity to rice fields and high temperatures. The seasonality of malaria was discussed in key informant interviews. For more than half of the traditional healers, who were generally the least informed group in the qualitative study in terms of symptoms and aetiology of malaria, *djèkouadjo *was considered particularly important in the dry season due to the sun. In contrast, the majority of modern health care providers mentioned that the highest frequency of malaria occurs during the rainy season.

### Treatment of *djèkouadjo*

More than 95% of the household heads interviewed stated that they had suffered at least once from *djèkouadjo*, about a third within the last three months. Over the past six months, 73 (32%) of the respondents declared that they had sought treatment of *djèkouadjo *in modern health facilities once (n = 37, 16%), twice (n = 17, 7%), or more often (n = 19, 8%). As summarised in Table 2, *djèkouadjo *was primarily addressed at the family level (Zatta: 89%, Adibrobo: 59%). The importance of the wider family circle was confirmed in a cross-check question where the primary assistants in applying traditional treatments were stated to be relatives (Zatta: 52%, Adibrobo: 30%).

The treatments of *djèkouadjo *could be classified into two groups, namely modern and traditional. Regarding modern medicine, *djèkouadjo *is treated with analgesics and antipyretics such as aspirin and paracetamol along with antimalarials such as chloroquine and amodiaquine. Traditional medicine mentioned by schoolchildren consisted of herbal teas from the leaves and bark of acacias (*Cassia siamea*), leaves of neem (*Azadirachta indica*), guava (*Psidium guajava*) and papaya (*Carica papaya*). One schoolgirl in Zatta summarised treatment options as follows: "*When we have malaria, we use leaves of acacias to make herbal tea or enema. We also buy aspirin (paracetamol) and nivaquine (chloroquine) to make additional treatment*."

According to traditional healers, treatment of *djèkouadjo *required the avoidance of some food items, especially red tomatoes, red peppers, red soup and red cooking oil since red food is related to the sun (cause), as well as to yellow eyes and urine (symptoms). There seemed to be no conclusive pattern relating the perceived causes and the chosen treatment. As shown in Table 2, regardless of whether sun, mosquitoes or mystical reasons were put forth as the main causes of *djèkouadjo*, more than half of the respondents (n = 136; 59%) exclusively used traditional treatments, whereas traditional treatments were combined with modern methods by a third of the interviewees (n = 76; 33%). There was a similar pattern for the relationship of mentioned symptoms and the use of traditional medicine. Although people were aware of malaria-related symptoms and their association with mosquitoes, folk perceptions prevailed.

Regarding the relation between normative or "ideal" treatment and applied or "actual" treatment, a significant overlap of traditional and modern ideas was noted. The ideal treatment of *djèkouadjo *for many people included both plant-based and modern treatments, which was respected in the actual treatment. The "normative modernists" in both villages, i.e. those advocating modern treatment, applied in practice slightly less traditional treatments than the "normative traditionalists" (traditional treatment only: 60% *vs*. 75%; mixed treatment: 38% *vs*. 24%). An interesting observation was made in Zatta; 35 of the 156 respondents (22%) stated prayer as the treatment most frequently described, and showed a tendency to modernist treatment compared to respondents denying the treatment by prayer (traditional treatment only: 54% *vs*. 68%; mixed treatment: 43% *vs*. 32%).

Results from the qualitative data indicate a sequential treatment and confirm that a combination of modern and traditional treatments is used as observed in the household survey. At the onset of symptoms, *djèkouadjo *is treated at home. Care from traditional healers or professional health care providers is only sought if treatment at home fails. A nurse working in Zatta put it as follows: "*Most of the villagers treat malaria at home in the beginning, and only if it gets worse, do they go to the hospital. It is the same in my home*".

### Prevention of *djèkouadjo*

There was no link between perceived causes of malaria and the practice of prevention. The majority of respondents claimed to be using measures to avoid mosquito bites rather than malaria (Zatta: 71%, Adibrobo: 75%), even if they affirmed that mosquitoes are the cause of malaria (Zatta: 70%, Adibrobo: 50%) (Table 2). Modern preventive methods among the Baoulé of Zatta included insecticide sprays (38%), bed nets (12%) and fumigation with burning coils (4%). With the exception of bed nets, levels of preventive measures were even lower among the Agni (28%, 17% and 2%, respectively). That only a small proportion of people use bed nets is explained by their perceived high costs (stated by 73% of the respondents). Aside from modern prevention methods, a quarter of the villagers also used herbal teas and enema to prevent malaria. The large majority of key informants cited measures such as burning orange peel, the use of odorous herbal plants in the house and avoiding the sun. However, as articulated by a traditional healer in Adibrobo, prevention of malaria remains a difficult task: "*Prevention of malaria is impossible for people who have blood in their veins and who work on the field under the sun. Malaria is in the blood, it is inborn. When the blood begins to change into water [due to the sun], malaria manifests itself*."

Those who believe that malaria is caused by God also believed that it is impossible to avoid the illness as it is only God who can prevent people from getting it. Conversely, amongst those who believed that *djèkouadjo *is acquired because of their behaviour, helpful preventive measures include keeping the environment clean, removing vegetation and stagnant water in close proximity to their homes, as well as adequate disposal of waste. A few respondents also mentioned wearing clean clothes and avoiding excessive consumption of sweet foods.

### Socioeconomic stratification and malaria-related social indicators

Table 3 summarises the data from the cross-sectional survey, stratified by people's socioeconomic status for each study village separately. The data showed that the least poor individuals considered headache (CI = 0.047, SE = 0.010) and yellow eyes (CI = 0.061, SE = 0.013) more often as symptoms of malaria than the poorest people. The notion of mosquito bites was strongly linked to socioeconomic status. The least poor reported mosquitoes more frequently than their poorer peers (CI = 0.105, SE = 0.043), but the intermediate classes did not necessarily follow a linear trend, with the middle class (poor) having a lower frequency than their neighbouring strata. Conversely, the poorest more frequently mentioned the exposure to the sun as the main cause for malaria, although the statistical analysis only allows for a tendency (CI = -0.028, SE = 0.030), and the trend was not linear.

**Table 3:** Knowledge, belief and practices about *djèkouadjo *among household heads in Zatta and Adibrobo, central Côte d'Ivoire

Variable	Total	Wealth quintiles					CI	SE	*t*-test (CI)
					
		Poorest (n = 47)	Very poor (n = 46)	Poor (n = 49)	Less poor (n = 44)	Least poor (n = 46)			
Symptoms									
Fever	70	79	67	57	64	85	0.009	0.048	0.18
Loss of appetite	48	45	50	44	48	54	0.028	0.019	1.51
Headache	34	30	33	33	39	37	0.047	0.010	4.96*
Yellow eyes	32	28	28	33	39	35	0.061	0.013	4.68*
Yellow urine	28	30	24	33	30	26	-0.005	0.024	-0.21
Vomiting	13	17	13	6	18	9	-0.075	0.084	-0.89
Perceived cause of malaria									
Sun	74	79	67	84	77	61	-0.028	0.030	-0.92
Mosquitoes	65	47	67	53	68	89	0.105	0.043	2.44*
God	7	6	13	6.	7	2.	-0.171	0.124	-1.38
Sorcerer	6	4	13	6	5	0	-0.241	0.205	-1.17
Ancestor	3	2	7	2.	3	0	-0.263	0.198	-1.33
Modern treatment									
Pill	91	86	88	100	93	85	0.004	0.018	0.24
Injection	45	57	47	38	31	45	-0.073	0.045	-1.63
Perfusion	7	0	12	8	6	5	0.063	0.213	0.29
Drinking solution	4	0	6	8	0	4	0.069	0.229	0.30
Modern prevention									
Insecticide spray	39	20	37	33	50	58	0.183	0.056	3.29*
Bed net	16	7	7	13	24	30	0.324	0.048	6.80*
Fumigating coil	4	6	4	4	5	2	-0.154	0.071	-2.17*
Traditional treatment									
Herbal tea	48	55	54	41	57	35	-0.064	0.044	-1.48
Enema	31	23	35	33	32	33	0.040	0.040	1.02
Washings	8.	9	4	8	2	17	0.153	0.156	0.99
Traditional prevention									
Herbal tea	43	41	64	40	50	13	-0.136	0.122	-1.12
Enema	41	41	36	40	20	75	0.0960	0.111	0.87
Does not know	17	18	9.	30	30	0	-0.0650	0.2113	-0.31
Avoidance of modern medicine									
High cost	61	61	67	62	61	58	-0.015	0.011	-1.30
Treatment inefficient	16	15	16	17	20	11	-0.017	0.061	-0.27
Unfriendly staff	7	7	9	6	7	7	-0.021	0.029	-0.74
Avoidance of traditional medicine									
Lengthy healing process	17	19	15	18	9	24	0.016	0.082	0.20
High cost	6.	4	9	6	7	4	-0.021	0.086	-0.25
Treatment inefficient	3	0	9	2	5	2	0.003	0.241	0.01
Does not know	0	2	0	0	0	0	-0.797	0.180	-4.44
Actions against mosquitoes									
Removal of vegetation	60	52	65	49	61	71	0.045	0.030	1.54
Removal of stagnant water	46	44	47	37	48	58	0.051	0.033	1.55
Insecticide spray	24	13	30	16	36	24	0.096	0.082	1.16
Bush fire	8	9	6	6	11	7	0.008	0.060	0.13

The use of preventative measures was strongly influenced by socioeconomic status, and was related to the perceived cost of the method in question. Individuals from the wealthiest group more frequently cited prevention of malaria with comparatively expensive methods such as insecticide spray (CI = 0.183, SE = 0.056) and bed nets (CI = 0.324, SE = 0.048). Conversely, the poorest respondents used fumigating coils more often since they were perceived to be cheaper (CI = -0.154, SE = 0.071). Finally, there was a tendency for less poor people to mention environmental measures of protection against mosquito bites such as the removal of vegetation and of stagnant water bodies.

## Discussion

The present study aimed to enrich an existing database on clinical and entomological findings of malaria in two rural communities in central Côte d'Ivoire [5,6,19] with people's knowledge, attitudes, practices and beliefs and the socioeconomic status of a random sample of household heads. It is important to note that studies in sub-Saharan Africa have shown a variety of taxonomies relating to 'malaria'. As found in the present study, the local term *djèkouadjo *is used to refer to one or several symptoms which, taken together, approximate a clinical diagnosis of malaria. In this case, malaria was not seen as a new illness introduced by the formal health system as described by Adongo and colleagues [27]. Usually there is an adequate knowledge of symptoms caused by malaria in endemic areas, and people are well aware of the clinical manifestations of the disease [28].

The knowledge of symptoms and causes of malaria in the current study area was found to be sound. For example, white palms (symptom for anaemia), and yellow eyes and yellow urine (symptoms for jaundice) were recognized as symptoms of malaria by household heads and key informants. Similar findings have been reported before from Ghana [27-29], Mali [30] and Uganda [31].

However, the respondents in this study also believed in 'natural' (e.g. sun, sweet and fatty diet) and 'mystic' (e.g. God and ancestors) causes for malaria. These findings are similar to observations made elsewhere [10,27,32-35]. Belief in witchcraft was not necessarily seen as opposed to belief in natural cause. Both beliefs complemented each other [36]. This is illustrated by the treatment of complicated malaria which, when not cured rapidly with the first-line treatment, was attributed to supernatural causes.

Several studies in sub-Saharan Africa have demonstrated such a dichotomy in knowledge between folk and biomedical interpretations of malaria in terms of causal processes [10,11,37-39]. A possible explanation for multiple aetiologies is that people receive information on malaria, mosquitoes and bed net usage, but have not yet been able to link this biomedical information with their cultural knowledge. Although malaria education and bed net promotion may not change community knowledge rapidly [27], it was found that interviewees who attained a higher educational level clearly associated malaria transmission with mosquitoes. Education campaigns thus influenced local knowledge. Interviewees in Zatta had a somewhat better knowledge on how malaria is transmitted, when compared to Adibrobo, possibly due to the community-awareness campaign for the promotion of ITNs, which had been carried out prior to the cross-sectional survey reported here.

Treatment-seeking did neither differ between ethnic and religious groups, nor by education level. The first step of treatment-seeking was typically based on self-medication, and often included the combined use of traditional medicine (herbal tea, bathing or enema) and modern drugs (pharmaceutics, including analgesics and antimalarials). Seeking professional medical advice and treatment was delayed until it became clear that the illness could not be adequately managed at home. Almost two-third of the interviewees seemed to be "traditionalist", i.e. using traditional medicine, rather than "modernist" (one-third). Similar findings have recently been reported for north-eastern Nigeria [40]. The preference towards folk treatment rather than formal health services might be explained by the fact that health care sought by traditional healers is usually quite inexpensive [10]. In addition, traditional treatment is often provided through more flexible services and pricing systems, which are readily adapted to the clients' ability to pay [41]. As observed in different African settings, the use of preventive methods is dependent on people's socioeconomic status, which was proxied by using a household asset-based index [18,23,24]. Previous work has revealed that the poorest people are the least likely to benefit from health interventions and adequate service delivery as shown in studies carried out in Tanzania [15] and Côte d'Ivoire [18].

Surprisingly, there was no correlation between the perceived causes and the choice of treatment. Even when interviewees believed in the established biomedical aetiology of malaria, they used traditional medicine, sometimes unaccompanied by modern medicine. At the same time interviewees who thought *djèkouadjo *to be caused exclusively by the sun, or sorcerers, also used modern treatment. Sick people generally used both treatment types during one episode of malaria regardless of the perceived causes. This flexibility may be due to their intimacy with the disease and their confidence in their ability to self-medicate at little expenses. Malaria and the management of the disease appear to be part of people's everyday life. This may explain why the awareness of various modern and traditional treatments for malaria was elevated, similar to observations made elsewhere [35]. It follows that the use of multiple modalities of care-seeking behaviour was a common feature in both villages, which correlates with findings of previous studies in other African settings [10,28,42,43].

Interestingly, not only the preferred treatment and prevention methods, but also the perception of the aetiology and symptoms of malaria were linked to socioeconomic status. The main perceived cause for malaria was exposure to the sun, which was highly prevalent across socioeconomic strata. Additionally, the poorest believed least in mosquitoes as cause for *djèkouadio*. Regarding symptoms, the significant links between perception and socioeconomic status appeared controversial.

An important factor governing malaria mortality and morbidity in endemic settings is the lack or insufficient attention given to preventive measures with proven efficacy [7]. This might be due to prevailing folk perceptions of the disease and perceived low efficacies of preventive tools or inaccessibility due to high costs or other reasons. In this study, among interviewees who related the cause of malaria to mosquitoes, only few protected their houses with wire screening on doors and windows. Bed nets were used only by a small fraction of the population. Bed net usage was limited due to perceived high costs and discomfort when sleeping under a net. Subsidizing of bed nets is a common request by rural communities [44]. In the 1990s, low coverage of bed nets and other personal protective measures at the household level was common [29,45-48]. Often, bed nets are regarded as a tool primarily to combat the nuisance of mosquitoes [49], and are, therefore, used by adults rather than by children [27]. In this study, the use of ITNs and the relatively more expensive insecticide spray (when used continuously) was linearly and significantly linked to socioeconomic status, hence in contrast to recent findings reported from Gabon [12]. The difference between the poorest and the least poor concerning bed net usage has been reported before [16-18]. A common explanation is that poorer populations have less access to effective means of prevention and treatment because they have lower purchasing power and flexibility [16-18,44]. The low fraction of households using bed nets calls for continued health education, and improving access to this preventive tool at no or low costs, so that the poorest population segments can purchase and protect themselves with bed nets.

In summary, observed patterns were not as clear-cut as expected, but nevertheless show that socioeconomic status goes beyond determining care-seeking behaviour and choice of prevention method, and is also associated with the perceived causes and symptoms of *djèkouadio*.

## Conclusion

Findings reported here from a rural part in central Côte d'Ivoire suggest that even people with sound knowledge of causes and symptoms of malaria continue to use traditional treatments, while those with folk beliefs also rely on modern treatment. Although socioeconomic status and education attainment are important drivers of the epidemiology and control of malaria, the study revealed that more complex mechanisms are waxing and waning the decision-making process of people who live in malaria-endemic countries.

A better understanding of the social dynamics regulating access to household resources might help to explain differences in decision-making for malaria prevention and treatment. Obrist and colleagues [50] have pointed out that knowledge of malaria alone is not enough. Access to different resources such as financial or social capital significantly influences health-seeking practices. It is concluded that equitable access to resources at household, community and health system levels are essential. Innovative and community-based approaches are warranted that match health care services with poor people's needs and resources. At the same time, the health care delivery system needs to align its services accordingly, for instance, by making vouchers available for low socioeconomic status groups or by offering free or subsidized prevention and treatment services.

## Competing interests

The authors declare that they have no competing interests.

## Authors' contributions

CE implemented the study, analysed the data and drafted the manuscript. JU, ABT, BGK, EKN and BO designed the study, supported the implementation, assisted in the interpretation of the data and the drafting and revision of the manuscript. GR, CP and SG contributed to the analysis and interpretation of the data and the revision of the manuscript. OG and MT contributed to the design of the study and the revision of the manuscript. All authors read and approved the final manuscript.
